# Bis(2,4-dimethyl­pyridinium) tetra­bromido­mercurate(II)

**DOI:** 10.1107/S1600536812046788

**Published:** 2012-11-17

**Authors:** Rawhi Al-Far, Salim F. Haddad, Basem F. Ali

**Affiliations:** a Faculty of Science and IT, Al-Balqa’a Applied University, Salt, Jordan; b Department of Chemistry, The University of Jordan, Amman 11942, Jordan; c Department of Chemistry, Al al-Bayt University, Mafraq 25113, Jordan

## Abstract

The asymmetric unit of the title compound, (C_7_H_10_N)_2_[HgBr_4_], consists of one cation and one half-anion, bis­ected by a twofold rotation axis passing through the metal atom. The anion exhibits a distorted tetra­hedral arrangement about the Hg^II^ atom. In the crystal, the cations and anions are linked by N—H⋯Br hydrogen-bonding inter­actions along [010]. Cation–cation π–π stacking and Br⋯Br inter­molecular inter­actions are absent.

## Related literature

For inter­molecular inter­actions, see: Desiraju (1997 ▶). For related structures, see: Al-Far & Ali (2007 ▶); Ali & Al-Far (2007 ▶); Ali *et al.* (2008 ▶). For structures containing the [HgBr_4_]^2−^ anion, see: Gowda *et al.* (2009 ▶); Li *et al.* (2009 ▶). For standard bond lengths in the cation, see: Allen *et al.* (1987 ▶). 
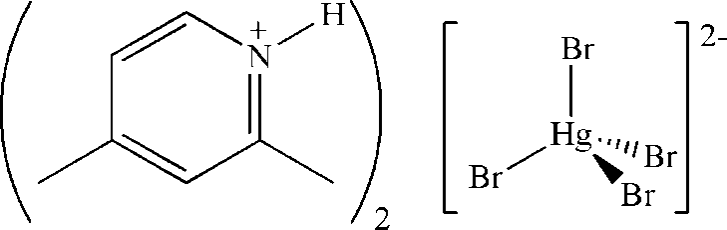


## Experimental

### 

#### Crystal data

(C_7_H_10_N)_2_[HgBr_4_]
*M* *_r_* = 736.51
Monoclinic, 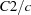

*a* = 20.022 (5) Å
*b* = 7.7985 (9) Å
*c* = 17.651 (3) Å
β = 129.12 (3)°
*V* = 2138.1 (11) Å^3^
*Z* = 4
Mo *K*α radiation
μ = 14.67 mm^−1^
*T* = 293 K
0.44 × 0.40 × 0.18 mm

#### Data collection

Agilent Xcalibur Eos diffractometer
Absorption correction: multi-scan (*CrysAlis PRO*; Agilent, 2011 ▶) *T*_min_ = 0.002, *T*_max_ = 0.072
5366 measured reflections
2895 independent reflections
1454 reflections with *I* > 2σ(*I*)
*R*_int_ = 0.036

#### Refinement

*R*[*F* ^2^ > 2σ(*F* ^2^)] = 0.050
*wR*(*F* ^2^) = 0.102
*S* = 1.01
2895 reflections
98 parameters
H-atom parameters constrained
Δρ_max_ = 1.45 e Å^−3^
Δρ_min_ = −1.54 e Å^−3^

### 

Data collection: *CrysAlis PRO* (Agilent, 2011 ▶); cell refinement: *CrysAlis PRO*; data reduction: *CrysAlis PRO*; program(s) used to solve structure: *SHELXTL* (Sheldrick, 2008 ▶); program(s) used to refine structure: *SHELXTL*; molecular graphics: *SHELXTL*; software used to prepare material for publication: *SHELXTL*.

## Supplementary Material

Crystal structure: contains datablock(s) I, global. DOI: 10.1107/S1600536812046788/bx2429sup1.cif

Structure factors: contains datablock(s) I. DOI: 10.1107/S1600536812046788/bx2429Isup2.hkl

Additional supplementary materials:  crystallographic information; 3D view; checkCIF report

## Figures and Tables

**Table 1:** Selected bond lengths (Å)

Hg1—Br1	2.5767 (11)
Hg1—Br2	2.6160 (11)

**Table 2:** Hydrogen-bond geometry (Å, °)

*D*—H⋯*A*	*D*—H	H⋯*A*	*D*⋯*A*	*D*—H⋯*A*
N1—H1*A*⋯Br2^i^	0.86	2.45	3.286 (7)	163

Symmetry code: (i) 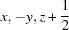.

**Fig. 1.**
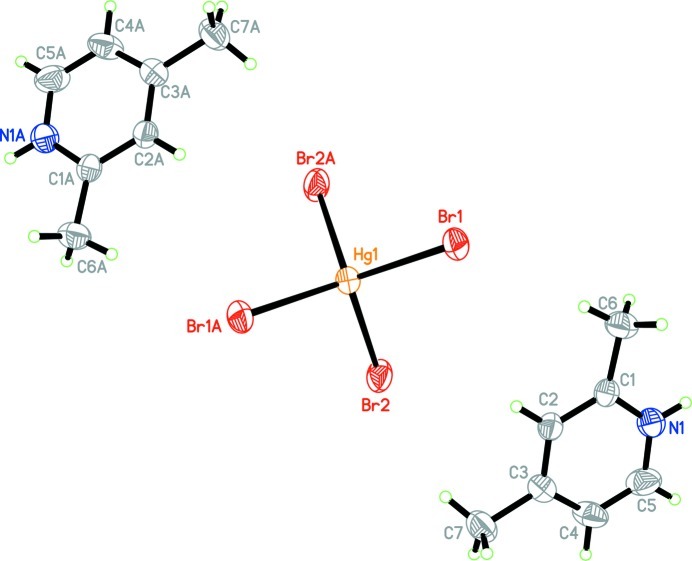
Molecular configuration and atom naming scheme for the title compound. Displacement ellipsoids are drawn at the 30% probability level. Symmetry operation (A) stands for -x + 1, y, -z + 3/2.

**Fig. 2.**
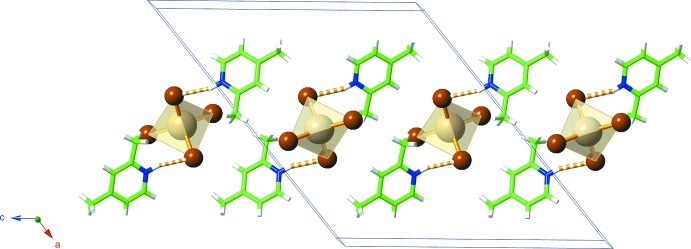
Packing diagram of the title compound, down crystallographic c axis. Interspecies hydrogen bonds are shown as dashed lines (N—HBr). Symmetry code (i) : x, -y, z+1/2.

## supplementary crystallographic information

### Comment

Noncovalent interactions play an important role in organizing structural units in both natural and artificial systems (Desiraju, 1997). In connection with ongoing studies (Al-Far & Ali 2007; Ali & Al-Far 2007; Ali *et al.*, 2008) of the structural aspects of bromometal anions salts, we herein report the crystal structure of the title compound, (I).

In the title compound, Fig. 1, the asymmetric unit of the title compound, (C_7_H_10_N)_2_[HgBr_4_], consists of one cation and one half-anion, bisected by a twofold rotation axis passing through the metal center. The anion exhibits a distorted tetrahedral arrangement about the Hg atom (Table 1). The Hg—Br1 and the symmetry related one [2.5767 (11)Å] bonds are almost invariant and significantly shorter than Hg—Br2 and symmetry related one [2.6160 (11)Å]. These lengths fall within the range of Hg—Br distances reported previously for compounds containing [HgBr_4_]^2-^ anions (Gowda *et al.*, 2009; Li *et al.* 2009). It is noteworthy that the longer Hg—Br2 and the symmetry related bonds are involved in more and shorter interactions than the shorter bonds (Table 1). In the cation, the bond lengths and angles are in accordance with normal values (Allen *et al.*, 1987). In the crystal structure the cations and anions are linked by N—H···Br hydrogen bonding interactions, Fig.2 along [010] direction. Cation···cation π···π stacking and Br···Br intermolecular interactions are absent.

### Experimental

A warm solution (40°C) of HgCl_2_ (1.0 mmol) dissolved in ethanol (10 ml; 95%), was added drop wise to a stirred hot solution of 2,4-dimethylpyridine (1 mmol) dissolved in ethanol (10 ml; 95%) and HBr (60%, 1 ml). During reflux for 2 h, liquid Br_2_ (1 ml) was added to the mixture. The final mixture was allowed to stand undisturbed at room temperature. Colorless crystals of the title salt formed in two days, filtered off and one crystal suitable for diffraction measurements is used to collect data.

### Refinement

All H atoms were positioned geometrically and refined using a riding model, with N—H = 0.86 Å and C—H = 0.93 and 0.96 Å, for aryl and methyl H-atoms, respectively. The *U*_iso_(H) were allowed at 1.5*U*_eq_(C methyl) or 1.2*U*_eq_(N/C non-methyl).

### Crystal data

**Table:**

(C_7_H_10_N)_2_[HgBr_4_]	*F*(000) = 1352
*M**_r_* = 736.51	*D*_x_ = 2.288 Mg m^−^^3^
Monoclinic, *C*2/*c*	Mo *K*α radiation, λ = 0.71073 Å
Hall symbol: -C 2yc	Cell parameters from 1281 reflections
*a* = 20.022 (5) Å	θ = 2.9–29.1°
*b* = 7.7985 (9) Å	µ = 14.67 mm^−^^1^
*c* = 17.651 (3) Å	*T* = 293 K
β = 129.12 (3)°	Block, colourless
*V* = 2138.1 (11) Å^3^	0.44 × 0.40 × 0.18 mm
*Z* = 4	

### Data collection

**Table:**

Agilent Xcalibur Eos diffractometer	2895 independent reflections
Radiation source: Enhance (Mo) X-ray Source	1454 reflections with *I* > 2σ(*I*)
Graphite monochromator	*R*_int_ = 0.036
Detector resolution: 16.0534 pixels mm^-1^	θ_max_ = 29.2°, θ_min_ = 2.9°
ω scans	*h* = −27→26
Absorption correction: multi-scan (*CrysAlis PRO*; Agilent, 2011)	*k* = −6→10
*T*_min_ = 0.002, *T*_max_ = 0.072	*l* = −24→16
5366 measured reflections	

### Refinement

**Table:**

Refinement on *F*^2^	Primary atom site location: structure-invariant direct methods
Least-squares matrix: full	Secondary atom site location: difference Fourier map
*R*[*F*^2^ > 2σ(*F*^2^)] = 0.050	Hydrogen site location: inferred from neighbouring sites
*wR*(*F*^2^) = 0.102	H-atom parameters constrained
*S* = 1.01	*w* = 1/[σ^2^(*F*_o_^2^) + (0.0354*P*)^2^] where *P* = (*F*_o_^2^ + 2*F*_c_^2^)/3
2895 reflections	(Δ/σ)_max_ < 0.001
98 parameters	Δρ_max_ = 1.45 e Å^−^^3^
0 restraints	Δρ_min_ = −1.54 e Å^−^^3^

### Special details

**Table:**

Geometry. All e.s.d.'s (except the e.s.d. in the dihedral angle between two l.s. planes) are estimated using the full covariance matrix. The cell e.s.d.'s are taken into account individually in the estimation of e.s.d.'s in distances, angles and torsion angles; correlations between e.s.d.'s in cell parameters are only used when they are defined by crystal symmetry. An approximate (isotropic) treatment of cell e.s.d.'s is used for estimating e.s.d.'s involving l.s. planes.
Refinement. Refinement of *F*^2^ against ALL reflections. The weighted *R*-factor *wR* and goodness of fit *S* are based on *F*^2^, conventional *R*-factors *R* are based on *F*, with *F* set to zero for negative *F*^2^. The threshold expression of *F*^2^ > σ(*F*^2^) is used only for calculating *R*-factors(gt) *etc*. and is not relevant to the choice of reflections for refinement. *R*-factors based on *F*^2^ are statistically about twice as large as those based on *F*, and *R*- factors based on ALL data will be even larger.

### Fractional atomic coordinates and isotropic or equivalent isotropic displacement parameters (Å^2^)

**Table:**

	*x*	*y*	*z*	*U*_iso_*/*U*_eq_	
Hg1	0.5000	0.17233 (6)	0.7500	0.0544 (2)	
Br1	0.54606 (6)	0.33656 (12)	0.90297 (6)	0.0647 (3)	
N1	0.3078 (5)	0.0541 (10)	0.9729 (5)	0.066 (2)	
H1A	0.3343	0.0396	1.0342	0.080*	
C1	0.3507 (5)	0.1249 (10)	0.9458 (6)	0.050 (2)	
Br2	0.36889 (6)	−0.01976 (14)	0.69426 (7)	0.0796 (4)	
C2	0.3064 (5)	0.1449 (10)	0.8472 (6)	0.053 (2)	
H2A	0.3348	0.1915	0.8260	0.064*	
C3	0.2217 (6)	0.0982 (11)	0.7793 (6)	0.054 (2)	
C4	0.1817 (6)	0.0263 (12)	0.8122 (7)	0.076 (3)	
H4A	0.1244	−0.0076	0.7677	0.091*	
C5	0.2249 (6)	0.0046 (14)	0.9087 (7)	0.086 (3)	
H5A	0.1976	−0.0443	0.9308	0.103*	
C6	0.4414 (6)	0.1759 (12)	1.0245 (6)	0.078 (3)	
H6A	0.4433	0.2624	1.0646	0.116*	
H6B	0.4658	0.2205	0.9961	0.116*	
H6C	0.4739	0.0777	1.0639	0.116*	
C7	0.1732 (6)	0.1298 (12)	0.6723 (6)	0.078 (3)	
H7A	0.1294	0.0438	0.6351	0.118*	
H7B	0.2122	0.1247	0.6581	0.118*	
H7C	0.1468	0.2411	0.6553	0.118*	

### Atomic displacement parameters (Å^2^)

**Table:**

	*U*^11^	*U*^22^	*U*^33^	*U*^12^	*U*^13^	*U*^23^
Hg1	0.0559 (3)	0.0639 (4)	0.0459 (3)	0.000	0.0334 (2)	0.000
Br1	0.0707 (6)	0.0762 (8)	0.0504 (5)	−0.0070 (5)	0.0398 (5)	−0.0130 (5)
N1	0.066 (5)	0.079 (6)	0.057 (5)	0.005 (4)	0.040 (4)	0.015 (4)
C1	0.058 (6)	0.044 (6)	0.055 (5)	0.001 (4)	0.039 (5)	−0.001 (4)
Br2	0.0820 (7)	0.1058 (9)	0.0702 (6)	−0.0375 (6)	0.0572 (6)	−0.0225 (6)
C2	0.057 (6)	0.056 (6)	0.053 (5)	−0.003 (4)	0.038 (5)	−0.002 (4)
C3	0.059 (6)	0.041 (5)	0.052 (5)	0.003 (4)	0.030 (5)	0.000 (4)
C4	0.050 (6)	0.084 (8)	0.065 (6)	−0.012 (5)	0.023 (5)	0.011 (6)
C5	0.058 (7)	0.111 (10)	0.087 (8)	0.005 (6)	0.046 (6)	0.029 (7)
C6	0.053 (6)	0.100 (9)	0.064 (6)	−0.012 (5)	0.030 (5)	−0.003 (6)
C7	0.078 (7)	0.087 (8)	0.053 (5)	0.013 (6)	0.033 (5)	0.010 (5)

### Geometric parameters (Å, º)

**Table:**

Hg1—Br1	2.5767 (11)	C3—C4	1.372 (12)
Hg1—Br1^i^	2.5767 (10)	C3—C7	1.502 (11)
Hg1—Br2	2.6160 (11)	C4—C5	1.349 (11)
Hg1—Br2^i^	2.6160 (11)	C4—H4A	0.9300
N1—C1	1.338 (9)	C5—H5A	0.9300
N1—C5	1.347 (10)	C6—H6A	0.9600
N1—H1A	0.8600	C6—H6B	0.9600
C1—C2	1.376 (10)	C6—H6C	0.9600
C1—C6	1.485 (11)	C7—H7A	0.9600
C2—C3	1.372 (10)	C7—H7B	0.9600
C2—H2A	0.9300	C7—H7C	0.9600
			
Br1—Hg1—Br1^i^	120.39 (5)	C5—C4—C3	120.4 (9)
Br1—Hg1—Br2	106.83 (4)	C5—C4—H4A	119.8
Br1^i^—Hg1—Br2	106.25 (5)	C3—C4—H4A	119.8
Br1—Hg1—Br2^i^	106.25 (5)	N1—C5—C4	119.6 (9)
Br1^i^—Hg1—Br2^i^	106.83 (4)	N1—C5—H5A	120.2
Br2—Hg1—Br2^i^	110.13 (6)	C4—C5—H5A	120.2
C1—N1—C5	123.1 (8)	C1—C6—H6A	109.5
C1—N1—H1A	118.5	C1—C6—H6B	109.5
C5—N1—H1A	118.5	H6A—C6—H6B	109.5
N1—C1—C2	116.9 (8)	C1—C6—H6C	109.5
N1—C1—C6	117.3 (8)	H6A—C6—H6C	109.5
C2—C1—C6	125.8 (8)	H6B—C6—H6C	109.5
C3—C2—C1	122.0 (8)	C3—C7—H7A	109.5
C3—C2—H2A	119.0	C3—C7—H7B	109.5
C1—C2—H2A	119.0	H7A—C7—H7B	109.5
C2—C3—C4	118.0 (8)	C3—C7—H7C	109.5
C2—C3—C7	121.2 (8)	H7A—C7—H7C	109.5
C4—C3—C7	120.8 (9)	H7B—C7—H7C	109.5
			
C5—N1—C1—C2	−0.5 (13)	C1—C2—C3—C7	176.6 (7)
C5—N1—C1—C6	179.1 (9)	C2—C3—C4—C5	0.6 (15)
N1—C1—C2—C3	1.3 (12)	C7—C3—C4—C5	−177.4 (9)
C6—C1—C2—C3	−178.3 (8)	C1—N1—C5—C4	−0.1 (15)
C1—C2—C3—C4	−1.4 (13)	C3—C4—C5—N1	0.1 (16)

Symmetry code: (i) −*x*+1, *y*, −*z*+3/2.

### Hydrogen-bond geometry (Å, º)

**Table:**

*D*—H···*A*	*D*—H	H···*A*	*D*···*A*	*D*—H···*A*
N1—H1*A*···Br2^ii^	0.86	2.45	3.286 (7)	163

Symmetry code: (ii) *x*, −*y*, *z*+1/2.

## References

[bb1] Agilent (2011). *CrysAlis PRO* Agilent Technologies, Yarnton, England.

[bb2] Al-Far, R. & Ali, B. F. (2007). *Acta Cryst.* C**63**, m137–m139. 10.1107/S0108270107006452 17413213

[bb3] Ali, B. F. & Al-Far, R. (2007). *Acta Cryst.* C**63**, m451–m453. 10.1107/S0108270107039856 17917215

[bb4] Ali, B. F., Al-Far, R. H. & Haddad, S. F. (2008). *Acta Cryst.* E**64**, m751–m752. 10.1107/S1600536808012336 PMC2961581 21202445

[bb5] Allen, F. H., Kennard, O., Watson, D. G., Brammer, L., Orpen, A. G. & Taylor, R. (1987). *J. Chem. Soc. Perkin Trans. 2*, pp. S1–19.

[bb6] Desiraju, G. R. (1997). *Chem. Commun.* pp. 1475–1482.

[bb7] Gowda, B. T., Foro, S., Terao, H. & Fuess, H. (2009). *Acta Cryst.* E**65**, m946. 10.1107/S160053680902772X PMC2977096 21583396

[bb8] Li, S.-J., Chen, A.-H., Zheng, Z.-Y., Liu, S.-W. & Liu, Q.-X. (2009). *Acta Cryst.* E**65**, m1652. 10.1107/S1600536809047461 PMC2971972 21578664

[bb9] Sheldrick, G. M. (2008). *Acta Cryst.* A**64**, 112–122. 10.1107/S0108767307043930 18156677

